# 

**Published:** 1849-12

**Authors:** 


The publication of the Transactions of the American Medical Association, which has been retarded by the delay of the Chairmen of Committees to furnish copies of their reports, is now in rapid progress, and the work will soon be issued. We look for its appearance with much interest.

